# Epidemiology, evolution, and future of the HIV/AIDS pandemic.

**DOI:** 10.3201/eid0707.017704

**Published:** 2001

**Authors:** B R Levin, J J Bull, F M Stewart

**Affiliations:** Emory University, Atlanta, Georgia 30322, USA. blevin@emory.edu

## Abstract

We used mathematical models to address several questions concerning the epidemiologic and evolutionary future of HIV/AIDS in human populations. Our analysis suggests that 1) when HIV first enters a human population, and for many subsequent years, the epidemic is driven by early transmissions, possibly occurring before donors have seroconverted to HIV-positive status; 2) new HIV infections in a subpopulation (risk group) may decline or level off due to the saturation of the susceptible hosts rather than to evolution of the virus or to the efficacy of intervention, education, and public health measures; 3) evolution in humans for resistance to HIV infection or for the infection to engender a lower death rate will require thousands of years and will be achieved only after vast numbers of persons die of AIDS; 4) evolution is unlikely to increase the virulence of HIV; and 5) if HIV chemotherapy reduces the transmissibility of the virus, treating individual patients can reduce the frequency of HIV infections and AIDS deaths in the general population.


### Epidemiology, Evolution, and Future of the HIV/AIDS Pandemic

**Bruce R. Levin,* J. J. Bull,† and Frank M. Stewart‡**
*Emory University, Atlanta, Georgia, USA; †University of Texas, Austin, Texas, USA; and ‡Brown University, Providence, Rhode Island, USA

We used mathematical models to address several questions concerning the epidemiologic and evolutionary future of HIV/AIDS in human populations. Our analysis suggests that 1) when HIV first enters a human population, and for many subsequent years, the epidemic is driven by early transmissions, possibly occurring before donors have seroconverted to HIV-positive status; 2) new HIV infections in a subpopulation (risk group) may decline or level off due to the saturation of the susceptible hosts rather than to evolution of the virus or to the efficacy of intervention, education, and public health measures; 3) evolution in humans for resistance to HIV infection or for the infection to engender a lower death rate will require thousands of years and will be achieved only after vast numbers of persons die of AIDS; 4) evolution is unlikely to increase the virulence of HIV; and 5) if HIV chemotherapy reduces the transmissibility of the virus, treating individual patients can reduce the frequency of HIV infections and AIDS deaths in the general population.

Of all the infectious diseases first recognized in the 20th century, AIDS has had not only the most profound effect on human illness and death, it ended the developed world's complacency about infectious diseases. Caused by HIV, AIDS is, as far as we know, always fatal, even with effective therapy. Within the past 50 to 100 years, HIV went from being maintained primarily, if not exclusively, in sooty mangabeys (HIV-2) and chimpanzees (HIV-1) (1-3) to being the etiologic agent of a worldwide pandemic. AIDS was not recognized as a specific disease until 1980, and HIV was not identified as the etiologic agent until 1983. Nevertheless, an estimated 16 million persons have died from AIDS worldwide with 50 million currently infected with HIV.

HIV exhibits considerable evolutionary potential and, with drug-resistant bacteria, may have done more to enhance widespread understanding of the importance of population and evolutionary biology to human health and medicine than any other example this past century. Although HIV was initially susceptible to a variety of drugs, resistance mutations have enabled the virus to skirt every drug in the biotech arsenal. In part because of this capacity for rapid evolution, developing an effective vaccine will be difficult.

In the study reported here, we used mathematical models to consider the epidemiologic and evolutionary future of the HIV/AIDS pandemic. We addressed four questions: 1) What factors contribute to the spread and limiting the spread of HIV/AIDS in human populations? 2) How long will it be before resistance to HIV infections and/or their pathology evolves in the human population? 3) Will evolution in the HIV-infected population favor an increase or decrease in the virulence of the virus? 4) What are the epidemiologic consequences of life-prolonging treatment on the incidence of HIV-infected persons and AIDS patients?

Address for correspondence: Bruce R. Levin, Department of Biology, Emory University, Atlanta, GA 30322, USA; fax: 404-727-2880; e-mail: blevin@emory.edu

### Age of Infection (AoI) Model

To consider the epidemiologic and evolutionary future of HIV/AIDS, we developed a mathematical model for the population dynamics of HIV/AIDS. Our model is based on those typically employed by demographers and actuaries (see reference 4 for our previous publication of it in a mathematical context). Changes in the numbers of persons infected are treated as a birth and death process; the "births" are new infections, and "death" is removal of infected hosts from the population. The course of an infection in an individual is characterized by (i) how many new infections it generates at each time interval (week) since that host was first infected (the equivalent of the birth rate), and (ii) the weekly likelihood of the removal of a host from the population (the death rate). By "age," we mean the "age of infection" (AoI)—the time in weeks since that host was first infected. Within this framework, an HIV infection has a life cycle different from that of most viral and other microparasitic infections, because the onset of the disease, AIDS, occurs long after the person is infected with HIV and the microparasite has started to proliferate. Although the passage through time and progression to disease is continuous, for tradition as well as convenience it is useful to characterize an HIV infection as having four distinct stages, which are described and given parameters in Table 1.

In the numerical (computer) simulation used here, infected persons pass through the HIV/AIDS gauntlet on a weekly basis. Each week throughout stage i of the infection they cause $R_i/L_i$ new infections (R = new infections; L = duration in weeks); these newly infected hosts then enter the gauntlet. (Note that the rate of transmission during a stage thus depends both on $R_i$ and $L_i$, not just on $R_i$.) Each infected host continues to progress through the different stages and transmit the virus until the final week of the third stage, $L_3$, when the infected host dies. For more details

Table 1. Stages in the age of infection (AoI) model used in this investigation

| Stage | Characteristics | Duration (weeks) | New infections[a] | Death rate |
|---|---|---|---|---|
| 0 | Establishment: virus enters and colonizes host | $L_0$ | 0 | 0 |
| 1 | Primary infection: virus proliferates to high densities; the host seroconverts, which causes an abrupt decline in the titer of circulating virus | $L_1$ | $R_1$ | 0 |
| 2 | Asymptomatic: circulating virus remains at low levels, and disease symptoms are absent | $L_2$ | $R_2$ | 0 |
| 3 | AIDS | $L_3$ | $R_3$ | 100% at end |

[a]Total number of new infections produced by persons in this stage.

about the model see (4). Copies of the FORTRAN 77 program used for the numerical results presented here can be obtained from Bruce Levin.

### HIV Dynamics

When an infectious disease is first introduced into a population, it has the greatest opportunity to spread because all hosts are susceptible. Thus, if we introduce a single infected person into a wholly susceptible population, the maximum opportunity exists for producing secondary infections, the number of which is traditionally designated as $R_0$ (1). Although $R_0$ is a measure of the potential of a disease to spread in a population, it not a measure of the rate at which that disease will spread. One way to measure the rate at which a disease spreads in a wholly susceptible host population is that used by demographers to represent the geometric expansion rate of populations, the intrinsic rate of increase, r . N persons at time 0 become $Ne^{rt}$ persons at time t, where e is the base of the natural logarithm (if N were an amount of money, r would be the compound interest rate; in this case, r is the intrinsic rate of increase in the number of infected persons). With age structure in the model, a certain "settling out" period occurs in which the ratios of numbers of infections at different stages oscillate. As these oscillations decay, r approaches its steady state value, which can be calculated from the rate of change in any of the age categories of the AoI distribution.

The long duration of infection is important in understanding the intrinsic rate of increase of HIV and its dissemination through a population. During the epidemic phase of the disease, when there are many susceptible hosts and the number of new infections is increasing geometrically, the contribution of transmissions occurring at later stages of the infection to the spread of the virus is severely discounted (4, 5). Thus, new infections transmitted by recently infected persons, in stage 1, contribute much more to the spread of HIV than infections from persons in stage 3 (12 years later).
To illustrate this principle, let us use the AoI distribution employed in our original study (4) and assume that all transmission of the virus is confined to just one of the four stages. A rate of increase of HIV of 0.50 per year (HIV infections doubling every 1.4 years) would require (a) 1.1 secondary infections if transmission occurred solely in stage 1 (between weeks 6 and 12 after the host is infected in our example); (b) 5.2 secondary infections if transmission occurred solely during the asymptomatic period (an average of 10 years in our example); and (c) 72 secondary infections if transmission occurred solely during the period after the onset of AIDS (at an AoI between 10 and 12 years in our examples. In perhaps more familiar terms, we can assume a direct analogy between these results and the concept of compound interest; models for the spread of disease in a population are formally analogous to economic models for the growth of money in an account. An interest rate of 1% per day, compounded daily, yields an annual rate of 3,800%. In a similar manner, new infections produced during stage 1 compound themselves many times within the 10-year period during the advance to AIDS. One implication of this result is that when HIV first enters a naïve population, if transmission occurs within the first month of infection, this early transmission will drive the epidemic. Using a different model and a closer tie to real data, Jacquez, Koopman and colleagues made a similar argument that early transmission is important in driving the epidemic (6, 7). This conclusion has a number of implications, the most immediately practical of which is that public health and education procedures to control the epidemic will fail if they are based on using serologic test results to identify infected persons (6). Infected persons may well have transmitted the virus before they seroconverted.

### Factors Limiting the Rate of Spread of HIV/AIDS

What limits the rate at which HIV spreads through a population? Although at least 50 million persons are infected with HIV, the human population (more than 6 billion persons) consists almost entirely of uninfected persons, and the global rate of increase in new HIV infections does not appear to have abated. However, unlike the case with influenza and measles, considerable geographic and cultural variation exists in the epidemiology of HIV/AIDS. In effect, the HIV pandemic has been largely restricted to subpopulations—risk groups within which the likelihood of infection is substantially greater than that in the population at large, e.g., gay men, injection drug users, and sex workers, their patrons, and their spouses (or other sex partners).

It seems reasonable as well as hopeful to expect that the rate of increase in new HIV infections will decline in a number of different populations. What processes can account for these declines in the incidence of new HIV infections and reductions in the rate of spread of this virus? Do they reflect the efficacy of public health measures and education programs leading to more prudent sexual and needle use behavior? Has chemotherapy reduced the transmissibility of the virus? Is evolution making these viruses less transmissible or humans less susceptible to HIV infections or both? Although it would be difficult to reject the possibility of these different factors contributing to reductions in the rate at which new HIV infections are increasing, it may well be that the dominant reason for observed declines in the rate of spread of this retrovirus lies in the progression (and confinement) of the epidemic in particular subpopulations (risk groups). The reductions in the spread of HIV in these subpopulations could be due to the saturation of the pool of susceptible hosts in these groups rather than to successful intervention or behavioral changes (see 7).

To illustrate the effect of the saturation of susceptible hosts in risk groups, we can consider a single AoI distribution in which the duration of the four different stages ($L_0$-$L_3$) are, respectively,4, 6, 520, and 104 weeks. During each of the latter three stages, in a wholly susceptible population, each infected person produces one secondary infection, $R_{01} = R_{02} = R_{03} = 1$.

As the infection spreads, fewer susceptible persons exist, and the number of secondary infections caused by each infected individual will be somewhat less than the maximum rate. We assume that the realized rates of transmission of HIV during each stage of the infection ($R_1$, $R_2$, and $R_3$) decline at a rate proportional to their respective maximum rates and the fraction of the population that is susceptible to the infection. For example, at an given time, t, $R_1(t) = R_{01}S(t)/N$, where S(t) is the number of susceptible hosts at time t and N is the total number of persons in that population, which is held constant. Since we are assuming that AIDS is the only cause of death, to maintain N, a susceptible host replaces each person that dies of AIDS.

Figure 1A shows how the densities of susceptible hosts, HIV-positive persons without AIDS, and persons with AIDS change over the course of time in a population with an initial number of $10^4$ susceptible hosts and two HIV-positive persons at the earliest age of the infection (week 1). The virus rapidly spreads through the host population who exhibit no sign of AIDS for the first 10 years. By the time the first AIDS cases are recognized, more than half of the original population of 10,000 hosts are infected with the virus. Because of the relative dearth of susceptible hosts, the rate of spread of HIV to new hosts has already declined. Eventually, equilibrium is achieved and the infection maintains a steady state. In this endemic phase, the densities of susceptible hosts, HIV-positive hosts not manifesting the symptoms of AIDS, and AIDS patients level off. With these parameters, this endemic phase is reached in about 30 years.

A historical interpretation of this result is that by the time HIV infection was recognized as a specific disease in the gay male populations of San Francisco, Los Angeles, and New York in the early 1980s, a substantial proportion of persons in those subpopulations, were already infected with the virus (6). Moreover, by that time, HIV/AIDS may have already been approaching its endemic phase in these risk groups. The rate at which endemic phase is approached as well as the frequency of HIV-positive persons and AIDS patients within a subpopulation depends on the absolute rate of transmission. This is illustrated in Figure 1B. The parameters used for generating this figure are identical to those in Figure 1A, except for the maximum rates of increase, which have been reduced by a factor of two, $R_{01} = R_{02} = R_{03} = 0.50$.

As a consequence of this lower rate of transmission, the endemic phase is not reached for more than 100 years, and the proportion of the population that is HIV-positive and has AIDS is markedly reduced.

The simple explanation of these results is that an epidemic cannot continue forever because the number of uninfected hosts eventually declines, which stops the expansion of infections. At equilibrium, the fraction of infected versus uninfected hosts depends on various parameters that are subsumed in the $R_{0i}$s of our model. Using condoms, reducing the numbers of sexual partners, providing sterile needles for injection drug users, and any other factor that reduces the likelihood of transmission of the virus would further reduce the fraction of the subpopulation infected with HIV. Also affecting the rate of spread of the disease would be the rate at which susceptible hosts enter a risk group. We hope this rate can be reduced by education.

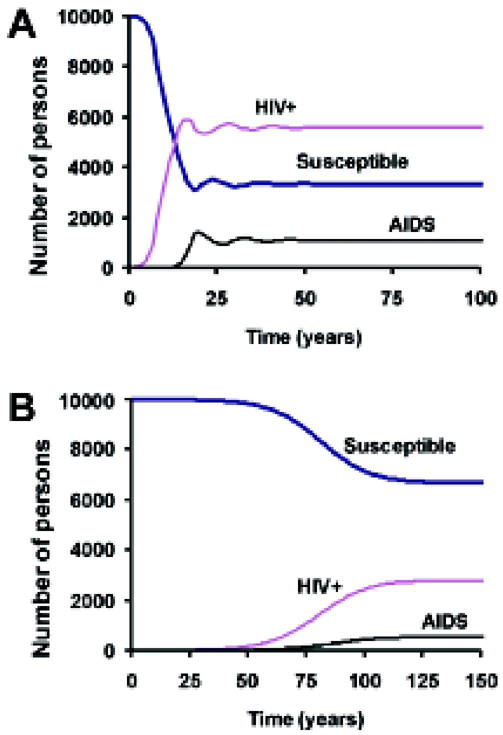

Figure 1. The spread of HIV/AIDS in a steady population of 10,000. In this figure, HIV-positive includes all persons infected with this virus, but not manifesting the symptoms of AIDS. In this simulation, the four stages of the infection, 0, 1, 2, and 3 are, respectively, 4, 6, 520, and 104 weeks ($L_0 = 4$, $L_1 = 6$, $L_2 = 520$, and L3 = 104). We assume that HIV is not transmitted during the first 4 weeks, stage 0. A) In a wholly susceptible host population during each stage 1, 2, and 3, infected host will be responsible for one secondary infection, $R_{01} = 0$, $R_{01} = R_{02} = R_{03} = 1.0$. B) In a wholly susceptible host population during stages 1, 2, and 3, each infected host will be responsible for one secondary infection, $R_{01} = 0$, $R_{01} = R_{02} = R_{03} = 0.50$.

### Evolution

Evolution in the human population could ultimately reduce the likelihood of becoming infected with a microparasite or of acquiring the disease if infected. Such changes in the host population could also impact the epidemiology and evolution of that microparasite. In this section, we describe simple models for human evolution in response to HIV and evolution of HIV's virulence in HIV-infected persons. We argue that it will take thousands of years before evolution in the human population substantially increases the fraction of persons resistant to HIV/AIDS. Evolution in the HIV-infected population at large, on the other hand, can proceed at an extremely high rate. On epidemiologic grounds, it is unlikely that evolution in this virus will make it more virulent (reduce the time between infection and the onset of AIDS) and may, in fact, favor reductions in virulence.

### Host Evolution

As long as an infectious disease causes some persons to die before or during reproductive years or to otherwise reduce the number of children they produce, natural selection will favor persons who are less susceptible to the infection and its deleterious effects. In the case of HIV, some evidence exists for inherited variation in the likelihood of HIV infection and in the rate of progression to AIDS among HIV-infected persons (8, 9). On the other hand, even under optimal conditions for rapid evolution—disease resistance is complete and determined by the genotype at a single locus—if the resistance gene is initially rare, it will take millennia before a substantial fraction of the population is of the resistant genotype.

This slowness of human evolution is illustrated in Table 2, in which we calculate the number of years required for a gene that confers a 10% advantage on a favored gene to reach a frequency of 0.50 for different initial frequencies of that gene and different modes of inheritance. For this calculation, we use the standard population genetics model for selection in a diploid population (10) and assume an average generation time of 20 years. In the case of infectious disease, the intensity of selection depends on the incidence of the disease as well as its effect on the fitness of infected hosts, so a 10% advantage for resistance could represent 20% of the population infected and a 50% loss of fecundity per infection, and so on. Thus, even if the genetic conditions for selection for resistance to AIDS were optimal, and the fertility of infected persons was substantially reduced, the intensity of selection for the resistant genotype would be no greater than the frequency of the infection in the population. Although in some populations the frequency of HIV infections is tragically and appallingly high, in the human population at large that frequency remains substantially less than 1%. Moreover, HIV-infected persons do produce viable uninfected children. The implications of this are straightforward if not optimistic: we cannot count on evolution in our population to save us from the AIDS epidemic, at least not in our lifetimes or that of many generations to come. On the other hand, in some areas, sub-Saharan Africa in particular, the incidence of the disease in the heterosexual population is so high that the intensity of selection for resistance would be considerably greater than 1%. If genes for resistance to HIV were present in the African population, resistance may in fact become common in sub-Saharan Africa more rapidly than in the human population at large. In any event, many persons will die of AIDS during the evolution process (11).

Table 2. Years before the frequency of a gene that confers a 10% advantage[a] to reach 0.50

| Initial frequency | Mode of inheritance: No dominance | Dominant | Recessive |
|---|---|---|---|
| 0.01 | 1,838 | 1,054 | 41,038 |
| 0.001 | 2,763 | 1,964 | 401,963 |
| 0.0001 | 3,684 | 2,884 | 4,002,884 |

[a]We are assuming that favored genotype has a 10% advantage over the other genotypes; in the no dominance case, the relative fitness of the heterozygote is intermediate between that of the two homozygotes. With a 1% selective advantage, it would take 10 times as long for the gene to reach a frequency of 50%.

### HIV Evolution of Virulence

Although human evolution is slow by our standards, HIV evolution will likely be rapid. Indeed, this retrovirus has already demonstrated its capacity for rapid evolution on several fronts, for example, the development of drug resistance and the ability to avoid the immune system. There is every reason to expect that HIV could evolve to a form with a different level of virulence in human hosts. Not so clear, however, is whether natural selection will favor changes in the virulence of this retrovirus or, if so, in what the direction that change would be. To predict the direction of natural selection on the virulence of HIV, we have to know the relationship between the virulence of this virus and its capacity for infectious transmission. Will HIV variants that engender a higher rate of progression to AIDS also be more transmissible and thus have an advantage over HIV variants that engender a lower rate of progression? Although a positive relationship between the transmissibility of HIV and its virulence has been proposed (12), no evidence supports this interpretation. Indeed, theoretical studies of the mechanisms of HIV virulence and experimental studies with simian retrovirus $SIV_{SM}$, which is almost identical to HIV-2, have found no evidence of a relationship between progression to AIDS and viral load or of a positive relationship between the transmissibility and virulence of HIV (13-19; M. Feinberg and S. Staprans, pers. comm.). Models of the epidemiology of HIV/AIDS can be used to elucidate how natural selection will operate on the virulence of HIV under different assumptions about the rate of progression to AIDS and the transmissibility of the virus.

Towards this end, we used our AoI model to explore how natural selection will operate in populations of humans infected with HIV who have different rates of progression to AIDS as measured by the length of the asymptomatic period. We made the simple and plausible assumption that transmission rates are constant within each stage of the infection and across different viruses, but that the total number of transmissions over the course of the infection varies only with the length of the stage of infection, $L_i$. This assumption constitutes a relationship between the virulence of this virus and its capacity for infectious transmission in a direction opposite from that assumed in (12), in that an earlier onset of AIDS is associated with fewer total transmissions from an infection.

Contrary to what may be anticipated from equilibrium considerations, with this model, a strain with a lower net yield of secondary infections can, under some conditions, have a selective advantage over a more productive strain. More specifically, if virulence is associated with a greater rate of transmission early in the infection, during the epidemic phase of the disease it could be favored, even if the overall transmission rate is reduced due to the earlier death of the infected host. This, too, is a manifestation of the advantages of early and discounting late transmission. On the other hand, as the disease approaches the endemic phase, the total amount of transmission over the term of the infection becomes increasingly important. During that stage, more virulent strains will be at a disadvantage unless they also have a higher overall rate of transmission.

To illustrate these points about the relationship between the epidemiology of the disease and the direction of selection for and against virulence, we used the AoI model to consider two distributions based on different lengths of time in the asymptomatic phase. The AoI distribution for the more virulent strain is characterized by parameters denoted with an asterisk (*) and that of the less virulent strain is the same as in Figure 1 (the asymptomatic period lasts 10 years). For the more virulent strain, the asymptomatic phase is 5 years ($L_2 = 10$, $L_2^* = 5$). The weekly rate of transmission within each stage is the same for both variants, but the more virulant variant experiences a shorter infection life span and thus produces proportionally fewer secondary infections, than the less virulent strain ($R_{02} = 1.0$, $R_{02}^* = 0.5$).

Figure 2A plots the changes in the total density of susceptible persons, HIV-positive persons, and persons with AIDS and the relative frequency of the more virulent virus. The frequency of the more virulent strain increases initially due to its early progression to AIDS and the consequent higher weekly rate of transmission during that stage. As the epidemic wanes and the endemic phase approaches, the frequency of this more virulent strain declines because it produces fewer secondary infections over the lifetime of the infection. Thus in this case, selection temporarily favors an increase in the virulence of HIV, but over the long term, reductions in the virulence of HIV will be favored.

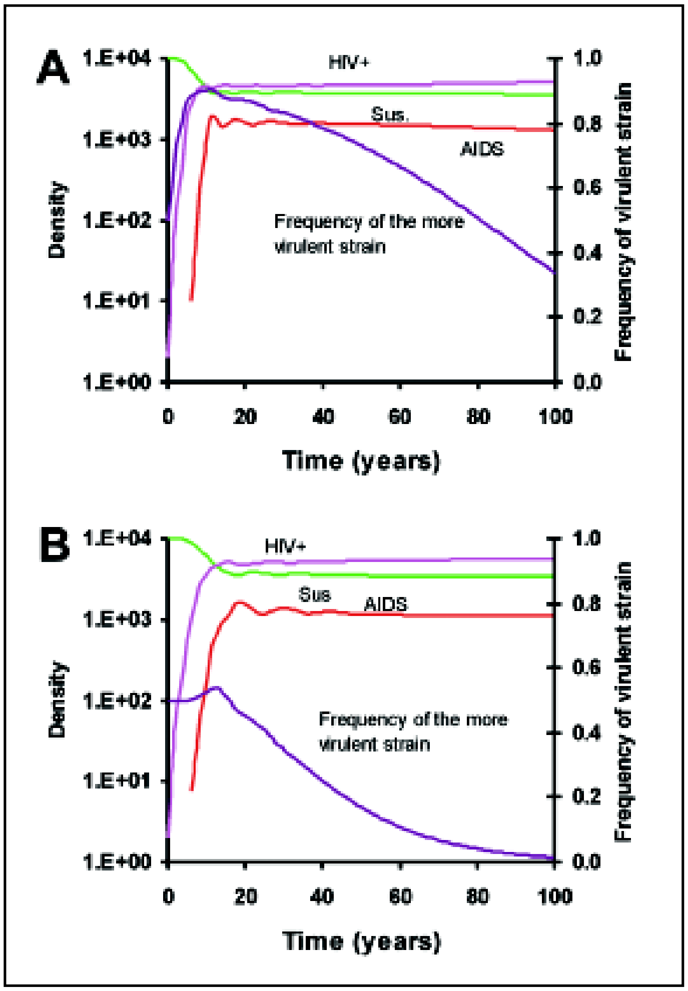

Figure 2. Evolution of HIV virulence in a steady state host population of 10,000. There are two HIV strains in these simulations. For one, the asymptomatic periods is 520 weeks and, in the more virulent population (*), it is 260 weeks. The duration of stages 0,1, and 3 is identical for both populations and the same as those in Figure 1. A) The weekly rates of transmission of the virus are identical in both populations, so that during the asymptomatic period in a wholly susceptible host population, the more virulent strain of HIV is responsible for 0.5 rather than 1 secondary infection, $R_{02}^* = 0.5$, while $R_{01} = 1.0$. The remaining $R_0$s of these two populations are identical to those in Figure 1. B) Early transmission in the more virulent strain is greater than that in the less virulent, $R_{01}^* = 1.05$, while $R_{01} = 1.0$ secondary infections. The weekly rates of transmission of are same for rest of the stages, $R_{00}^* = R_{00} = 0$, $R_{02}^* = 0.5$, $R_{02} = 1$, and $R_{03}^* = R_{03} = 1$.

If, for physiologic reasons, a faster progression to AIDS (stage 3) is associated with a higher absolute rate of transmission during earlier stages, during the epidemic phase of the disease, the rate of increase of the more virulent strain would be greater. Also greater would be the frequency before onset of the endemic phase and the intensity of selection against virulence (compare Figures 2A and 2B). Stated another way, even if there were a direct association between transmission and virulence during the early stage of the infection, virulence would eventually be selected against as the virus became endemic, provided that the total number of transmissions is lower for the more virulent strain.

Nonetheless, one should interpret these results cautiously because the evidence that no relationship exists between the virulence of HIV and its transmissibility remains largely circumstantial, albeit more compelling than that for a positive relationship. Until the results of studies addressing this issue become unequivocal, we cannot rule on the plausibility of the different scenarios for evolution of increasing or decreasing virulence of this retrovirus.

### Epidemiologic Consequences of Treatment

It may be some time before we have vaccines that are effective in preventing HIV infections. On the other hand, multidrug chemotherapy substantially prolongs the life of HIV-infected persons. For those who can afford this relatively expensive therapy or otherwise have access to these drugs, multidrug chemotherapy has literally been a lifesaver. From an epidemiologic perspective, however, is there a downside to this therapy?

On first consideration, it seems obvious that if treated HIV-infected persons survive longer and continue to transmit the virus at the same rates as they would have without chemotherapy, the virus will spread more rapidly than it would in the absence of treatment. This "perverse" effect of therapy was in fact explained nearly 10 years ago in a theoretical study by Anderson, Gupta and May (20). That research was based on a compartment model that was more specific about the mode of transmission than is our AoI model, but it did not take into account either the AoI distribution or reductions in transmission rates due to the limitation of susceptible hosts. They concluded "that in communities where the transmission rate of HIV is low, but sufficient for long-term persistence ($R_0$ not much greater than unity), treatment that lengthens the infectious period is likely to be able to increase the overall transmission rate to more than counterbalance the greater longevity of infected persons who are treated." Anderson and his collaborators also concluded that when transmission rates are already high, community-wide treatment would benefit both the individual and the community.

We used the AoI model to explore this question of the effect of treatment on the epidemiology of AIDS. If we assume that treatment extends the survival time of AIDS patients and has no effect on the rate transmission, then our results are the same as those of Anderson and colleagues (20). Treatment can increase the rate at which persons become HIV-positive and later acquire AIDS. On the other hand, there is every reason to expect that anti-HIV chemotherapy will markedly reduce the density of HIV in serum and strong evidence that transmission rates are directly proportional to the density of HIV in serum. Indeed, the results of an impressive recent study of HIV transmission by Quinn and colleagues (21) suggest that transmission will not occur at all when the viral titers are <1,500 copies/ml. With successful multidrug HIV therapy, viral titers of that level and lower can be expected and sustained for some time during the course of treatment.

Thus, the question of concern now is—what are the effects of reduced transmission of HIV from treated patients on the epidemiology of HIV/AIDS? To address this question, we used our AoI model to explore the effects of chemotherapy on the fraction of HIV-positive persons (non-AIDS) and persons with AIDS in treated and untreated groups during the epidemic and endemic phases of the disease. We considered a situation in which the overall rate of transmission in untreated hosts is relatively low, $R_{00} = 0$, $R_{01} = R_{02} = R_{03} = 0.5$ when the negative epidemiologic consequences of treatment are anticipated to be most profound (20). We assumed that treatment would extend the time before a person manifests the symptoms of an HIV infection, AIDS, by a factor of three, from 2 to 6 years. Here, parameters for a treated host will be denoted with *; $L_3 = 104$ weeks, $L_3^* = 312$ weeks. In one case, we assumed that treatment has no effect on the total number of viruses transmitted by a person with AIDS, but that it reduces the weekly rate of transmission by a factor of three. That is, in the course of the threefold increase in survival time, treated persons would be responsible for as many secondary infections as untreated AIDS patients ($R_{03} = R_{03}^* = 0.5$). In the second case,we assumed that treatment reduces the overall transmission by persons with AIDS by a factor of two ($R_{03} = 0.5$, $R_{03}^* = 0.25$).

To illustrate the effect of treatment in these situations, we compare what happens to the incidence of HIV and AIDS in a population in which AIDS patients are treated with a corresponding population in which they are not. If treatment has no effect on the overall rate of transmission, extending the life of AIDS patients will have virtually no effect on the fraction of the population infected with HIV (Figure 3A). While the infection is in the endemic phase, treatment increases the fraction of the population with AIDS by a factor of three, primarily by increasing the lifespan of AIDS patients by that amount. If, as seems reasonable to expect, chemotherapy actually reduces the overall transmission by persons with AIDS (Figure 3B), its epidemiologic effects will be positive. The incidence of HIV infections will be markedly reduced, and not until later in the endemic phase will the proportion of the population with AIDS increase, and that will be due largely to extending the lifespan of AIDS patients.

### Conclusions, Caveats, and Recommendations

The results of this theoretical study and others have generated the following hypotheses, predictions, and speculations about the epidemiologic and evolutionary future of HIV/AIDS. 1) The AIDS epidemic has been driven primarily by transmission of the virus early in the course of infection. 2) Declines and leveling off in the incidence of new HIV infections in subpopulations (risk groups) could be largely due to a dearth of susceptible hosts in (or entering) the subpopulation rather than to the efficacy of public heath measures, education, and chemotherapy or to the evolution of the virus. 3) Although AIDS-mediated selection in the human population will eventually increase the overall level of resistance to HIV infection or reduce the rate (and maybe even the likelihood) of progression to AIDS, it will take millennia before human evolution alone will significantly increase our resistance to HIV/AIDS. 4) Epidemiologic considerations provide no reason to anticipate that evolution will increase the virulence of HIV. 5) In populations in which HIV is relatively rare, treatment that simultaneously extends the lifespan of persons with HIV, and also reduces the rate of transmission of the virus, can lead to substantial declines in the number of HIV-infected persons in the general population.

We have evaluated the possible consequences of different properties of HIV transmission and evolution. However, despite all that has been learned about HIV/AIDS, existing knowledge about the biology and epidemiology of this retrovirus is still too rudimentary to employ empirical estimates of these parameters. Thus, it is not yet possible to make robust, quantitative predictions about (and explanations for) the epidemic and endemic behavior of HIV or the evolution of its virulence. Towards these desired ends, however, we believe that the AoI model

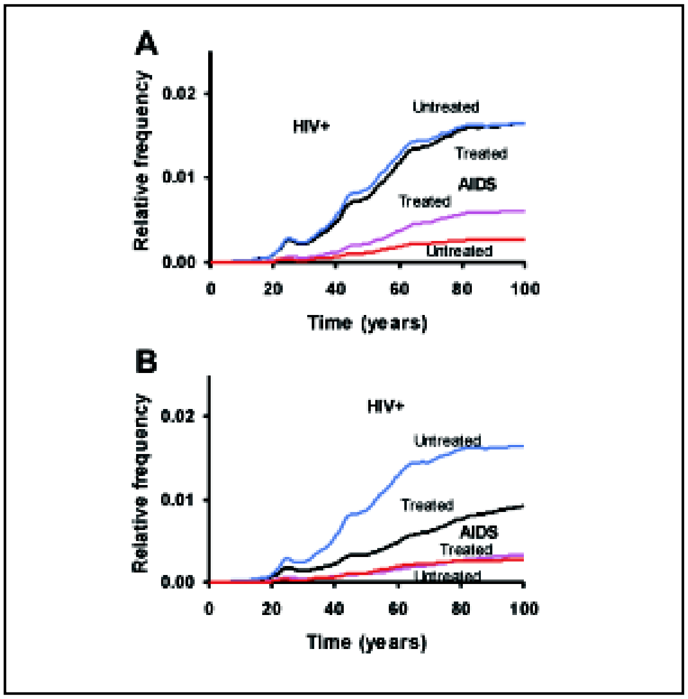

Figure 3. The effect of treatment on the relative frequency of HIV-positive persons (non-AIDS), and persons with AIDS in a steady state population of 10,000. In the untreated population, the duration of AIDS is 2 years, 104 weeks, before the patient dies, whereas in the treated population (*), it is 6 years, 312 weeks. The length of the other stages are identical to those in Figure 1. A) No effect of treatment on the overall transmission of HIV by AIDS patients, but a 1/3 reduction in the weekly rate of transmission. $R_{00} = R_{00}^* = 0$, $R_{01} = R_{01}^* = R_{02} = R_{02}^* = 0.5$, $R_{03} = 0.5$, $R_{03}^* = 0.5$. B) Treatment reduces the overall rate of transmission by AIDS patients by a factor of two. $R_{00} = R_{00}^* = 0$, $R_{01} = R_{01}^* = R_{02} = R_{02}^* = 0.5$, $R_{03} = 0.25$, $R_{03}^* = 0.25$

considered here and other mathematical models of the epidemiology of HIV serve the important role of revealing which properties of infections with this retrovirus and transmission are critical to understanding how it spreads, how to control that spread, and what to look for to predict the direction of evolution of its virulence.

Even without precise estimates of the values of these parameters, theoretical studies of the epidemiology of HIV make a number of unequivocal predictions. One is that early transmission will dominate the spread of HIV in naive populations. Another is that in populations in which HIV is relatively rare, treatment that does not reduce transmission rates can exacerbate the epidemic, and treatment that does reduce transmission can benefit the population as well as the patient. A broader, more definitive, and more quantitatively precise set of predictions about the epidemiologic and evolutionary future of HIV/AIDS will require data addressing the following questions. What are the rates of transmission of HIV during different stages of the infection? What effect does multidrug therapy have on the rate at which this virus is transmitted? Also critical to predicting the future HIV/AIDS is an objective and quantitative assessment of the demographic, behavioral, medical, and other reasons for changes in the incidence of HIV infections in different subpopulations. Are the declines in the rate of new HIV infections due to the efficacy of public health and education measures or, as suggested here, are they due to the saturation of the susceptible hosts in that risk group? Finally, to formally address the question of how HIV evolution will affect virulence of this retrovirus, we must know how much of the variation in the rate of progression to AIDS can be attributed to variation in the HIV-infected population.

Such data are not easy to obtain. Indeed, the potential importance of early HIV transmission (before seroconversion) was identified nearly a decade ago, yet little data have been collected on the magnitude of early transmission or on the amount of transmission occurring during other stages of the infection. From the narrow perspective of funding and careers, embarking on a research program directed at the acquisition of such data may be unwise. Gathering those kinds of data certainly lacks the romance and appeal of vaccine and drug development or the yield of generating more data on sequence variation. Nevertheless, without these transmission data, predictions about the epidemiologic and evolutionary future of HIV/AIDS will have to be relegated entirely (and, we believe, unsatisfactorily) to mathematical modeling.

### Acknowledgments

We thank Mark Feinberg, Ira Longini, and Sylvija Staprans not only for their useful comments on earlier drafts but also for providing us with information we should have known and for, in some cases, setting us straight.

This endeavor received support from the following research grants: NIH GM33782 (BRL), AI40662 (BRL), GM 57756 (JJB), the NSF DEB9726902(JJB), and TIAA/CREF (FMS).

Dr. Levin is professor of biology at Emory University, where he conducts theoretical and experimental research on the population biology and evolution of bacteria, infectious disease, and drug resistance.

### References

1. Gao F, Bailes E, Robertson DL, Chen Y, Rodenburg CM, Michael SF, et al. Origin of HIV-1 in the chimpanzee *Pan troglodytes troglodytes*. Nature 1999;397:436-41.
2. Korber B, Muldoon M, Theiler J, Gao F, Gupta R, Lapedes A, et al. Timing the ancestor of the HIV-1 pandemic strains. Science 2000;288:1789-96.
3. Sharp PM, Bailes E, Robertson DL, Gao F, Hahn BH. Origins and evolution of AIDS viruses. Biol Bull 1999;196:338-42.
4. Levin BR, Bull JJ, Stewart FM. The intrinsic rate of increase of HIV/AIDS: epidemiologic and evolutionary implications. Math Biosci 1996;132:69-96.
5. May RM, Anderson RM. Parasite-host coevolution. Parasitology 1990;100:S89-101.
6. Koopman JS, Jacquez JA, Welch GW, Simon CP, Foxman B, Pollock SM, et al. The role of the primary infection in epidemics of HIV infection in gay cohorts [see comments]. J Acquir Immune Defic Syndr 1997;7:1169-84.
7. Koopman JS, Jacquez JA, Welch GW, Simon CP, Foxman B, Pollock SM, et al. The role of early HIV infection in the spread of HIV through populations J Acquir Immune Defic Syndr 1997;14:249-58.
8. Hendel H, Henon N, Lebuanec H, Lachgar A, Poncelet H, Caillat-Zucman S, et al. Distinctive effects of CCR5, CCR2, and SDF1 genetic polymorphisms in AIDS progression. J Acquir Immune Defic Syndr 1998;19:381-6.
9. Weiser BH, Burger T, Charbonneau R, Grimson A, Visosky S, Nachman A, et al. CCR-5 genotype and resistance to mother-to-child transmission of HIV-1. HIV Pathog Treat Conf 1998;79:3081.
10. Crow JF, Kimura M. An introduction to population genetics theory. 1st ed. New York: Harper and Row; 1971.
11. Haldane J. The cost of natural selection. Journal of Genetics 1957;55:511-24.
12. Ewald PW. Transmission modes and the evolution of virulence, with special reference to cholera, influenza, and AIDS. Human Nature 1991;2:1-30.
13. Nowak MA, Anderson RM, McLean AR, Wolfs TF, Goudsmit J, May RM. Antigenic diversity thresholds and the development of AIDS. Science 1991;254:963-9.
14. Mittler J, Antia R, Levin B. Population dynamics of HIV pathogenesis. Trends in Ecology and Evolution 1995;10:224-7.
15. Frost SD, McLean AR. Germinal centre destruction as a major pathway of HIV pathogenesis. J Acquir Immune Defic Syndr 1994;7:236-44.
16. Bull, JJ. Virulence. Evolution 1994;48:1423-37.
17. Broussard SR, Staprans SI, Ray P, White R, Whitehead EM, Feinberg MB, et al. Simian immunodeficiency virus (SIVagm) replicates to high levels in naturally-infected African green monkeys without inducing immunologic or neurologic diseases. J Virol 2001;75:2262-75.
18. Stranford SA, Skurnick J, Louria D, Osmond D, Chang SY, Sninsky J, et al. Lack of infection in HIV-exposed persons is associated with a strong CD8(+) cell noncytotoxic anti-HIV response. Proc Natl Acad Sci U S A 1999;96:1030-5.
19. Levin BR, Bull J J. Short-sighted evolution and the virulence of pathogenic microorganisms. Trends Microbiol 1994;2:76-81.
20. Anderson RM, Gupta S, May RM. Potential of community-wide chemotherapy or immunotherapy to control the spread of HIV-1. Nature 1991;350:356-8.
21. Quinn T, Wawer M, Sewankambo N, Serwadda D, Li C, Wabwire-Magen F, et al. Viral load and heterosexual transmission of Human Immunodeficiency Virus type 1. N Engl J Med 2000;342:921-9.
